# Right to health and social justice in Bangladesh: ethical dilemmas and obligations of state and non-state actors to ensure health for urban poor

**DOI:** 10.1186/s12910-018-0285-2

**Published:** 2018-06-15

**Authors:** Sohana Shafique, Dipika S. Bhattacharyya, Iqbal Anwar, Alayne Adams

**Affiliations:** 1 Health Systems and Population Studies Division, icddr,b, Dhaka, Bangladesh; 2 Department of International Health, Georgetown University, Washington, DC USA

**Keywords:** Urban health, Global health, Ethics, Social justice, Health and human rights, Right to health, Social determinants of health, Urban poor, Bangladesh

## Abstract

**Background:**

The world is urbanizing rapidly; more than half the world’s population now lives in urban areas, leading to significant transition in lifestyles and social behaviours globally. While offering many advantages, urban environments also concentrate health risks and introduce health hazards for the poor. In Bangladesh, although many public policies are directed towards equity and protecting people’s rights, these are not comprehensively and inclusively applied in ways that prioritize the health rights of citizens. The country is thus facing many issues that raise moral and ethical concerns.

**Methods:**

A narrative literature review was conducted between October 2016 and November 2017 on issues related to social justice, health, and human rights in urban Bangladesh. The key questions discussed here are: i) ethical dilemmas and inclusion of the urban poor to pursue social justice; and ii) the ethical obligations and moral responsibilities of the state and non-state sectors in serving Bangladesh’s urban poor. Using a Rawlsian theory of equality of opportunity to ensure social justice, we identified key health-related ethical issues in the country’s rapidly changing urban landscape, especially among the poor.

**Results:**

We examined ethical dilemmas in Bangladesh’s health system through the rural–urban divide and the lack of coordination among implementing agencies. The unregulated profusion of the private sector and immoral practices of service providers result in high out-of-pocket expenditures for urban poor, leading to debt and further impoverishment. We also highlight policy and programmatic gaps, as well as entry points for safeguarding the right to health for Bangladeshi citizens.

**Conclusions:**

The urban health system in Bangladesh needs a reform in which state and non-state actors should work together, understanding and acknowledging their moral responsibilities for improving the health of the urban poor by engaging multiple sectors. The social determinants of health should be taken into account when formulating policies and programs to achieve universal health coverage and ensure social justice for the urban poor in Bangladesh.

## Background

As early as 1948, the World Health Organization (WHO), described health as “a state of complete physical, mental and social well-being and not merely the absence of disease or infirmity” [1]. The 1978 Alma-Ata Declaration further emphasized its broader social importance by conceiving of health as a “social goal whose realization requires the action of many other social and economic sectors in addition to the health sector” [1]. In short, equity in health and healthcare were firmly established as central to the pursuit of social justice [2, 3].

The notion of health as a human right and essential to ensuring human welfare and development [4] dates back to the Universal Declaration of Human Rights (UDHR) in 1948 [5] and the International Covenant on Economic, Social and Cultural Rights (ICESCR) in 1966 [6]. The ICESCR established particular objectives for improving health provision, which emphasized people’s right to access health services without discrimination and the State’s duty to provide essential medicines and ensure equitable distribution of health facilities, goods, and services [6].

Bangladesh is a signatory to most of these international declarations and ratified international agreements. The Constitution of Bangladesh also gives high priority to the development of the social sector, including health and education [7]. Constitutional provisions guarantee employment with reasonable wage, the right to social security and good quality of life, and the protection, promotion, and respect of healthcare as a constituent of human rights in Bangladesh [8]. In recent years, the country has witnessed extraordinary gains in health and has been applauded for its deliberate policy and programmatic focus on health equity [9]. Although many public policies in Bangladesh are directed towards equity and social justice, implementation tends to lack a comprehensive and inclusive approach that prioritizes the health rights of its citizens. This is particularly evident in the latest urban health survey, which revealed the existence of large-scale inequity and differentials in terms of health service use and health outcomes between slum and non-slum dwellers in the urban context of Bangladesh. [10] These discrepancies are even greater for people with disabilities and/or living in gender discriminating environments.

With rapid global urbanization, more than half of the world’s population currently lives in urban areas [11]. While city living offers many opportunities and services, urban environments also concentrate health risks and introduce health hazards and disadvantages for some segments of the population. Inadequate housing and food insecurity, coupled with lack of social protection, increase the burden of disease among the poor, especially those living in slums [11]. In addition, low-income urban dwellers are the most susceptible to natural disasters and the negative effects of unplanned and unregulated growth, such as increased air pollution, carbon emissions by industries, and unsafe work conditions leading to disability and death [12].

In the past few decades, the South Asian countries have experienced rapid development and urbanization, accompanied by widening inequalities in wealth and health [13]. In Bangladesh, the rate of migration into Dhaka and other urban areas [14, 15] continues to increase, driven by perceived economic opportunity and the negative impact of climate change on coastal livelihoods [16, 17]. Dhaka, the capital, has become the most densely populated city in the world [15], and over 30% of its residents, including female garment workers, live in urban slums, or on streets, rail stations, and railroad tracks. Studies have revealed that street dwellers remain out of the formal health service delivery mechanism and suffer continuously from various diseases [18]. It was also found that the formal health system of the Bangladesh government lacks adequate resources and support to address the vulnerability of pregnant garment workers in relation to the physical and mental stress they face in their workplace [19]. This heightened vulnerability and social exclusion [20] of this large segment of the population raise serious moral and ethical concerns.

In this paper, we explore issues related to social justice, health, and human rights in the context of rapid urban growth in Bangladesh and discuss the present situation from the right-to-health perspective. The key questions discussed here are: i) ethical dilemmas and inclusion of the urban poor with a view to achieving social justice; and ii) the ethical obligations and moral responsibilities of the state and non-state sectors in serving Bangladesh’s urban poor.

## Methods

A narrative review was carried out of the literature published in English and available in the databases of PubMed, Google Scholar, WHO, and the United Nations. In addition to these, manual searching was conducted to identify and review the relevant articles in the organizational database and library of International Centre for Diarrhoeal Disease Research (icddr,b). Articles covered a broad range that included philosophical debates, public health ethics, and descriptive reports, as well as qualitative and quantitative studies. The key words used were: “health and human rights”, “social justice”, “urban health”, “urban poor”, and “Bangladesh”. The literature search was conducted between October 2016 and November 2017; during this period the collected literature was reviewed and synthesized for analysis. The review involved two stages: we first conducted an extensive search of the existing literatures, and then we screened the collected literatures in terms of their relevance to issues of social justice. During the review process efforts were made to synthesize the relevant materials to gain a comprehensive understanding.

Applying the social justice theory of American philosopher John Rawls [21], we identified key health-related ethical issues in the country’s rapidly changing urban landscape, especially among the poor. In this paper, we begin by presenting a brief outline of Rawls’ theory of “equality of opportunity” to ensure social justice. We then illustrate Norman Daniels’ approach of extending Rawls’ theory into the arena of public health, applying that theory particularly to addressing the social determinants of health. Subsequently, we examine the ethical dilemmas and obligations related to healthcare in Bangladesh context, with special attention to poor and marginalized populations in urban areas. Finally, we discuss how Rawls’ theory could serve as a useful guide for addressing the ethical challenges of formulating and implementing policy decisions in Bangladesh to ensure equitable healthcare, with a focus on the most vulnerable population.

In his book *A Theory of Justice*, John Rawls describes “justice as fairness” and emphasizes the role justice needs to play within the social contract [21]. In his view, principles of social justice should guide how society organizes to enable the equitable distribution of resources. To ensure fair distribution, Rawls proposes the idea of “original position”—a hypothetical situation in which people imagine themselves in a blank state, where they know nothing about their place in society (based on class, gender, ethnicity, birthplace, socio-economic background, and other characteristics, etc.). This blank state, in which individuals are unaware of their social position, is termed “a veil of ignorance”. Rawls argues this veil of ignorance is important for formulating the set of principles that will keep society functioning effectively without undue unfairness to any particular individual or group. The equality principle seeks a level playing field so that a person’s social position—which is beyond an individual’s control—does not influence their outcomes [21].

The concept of social justice emphasizes the rational disbursement of common benefits and the sharing of collective problems. It encompasses two moral impulses that are relevant to public health: a) the improvement of human well-being by advancing health; and b) the obligation to focus on the health of the most underprivileged [21]. Extending Rawls’ theory of justice, Norman Daniels argued that justice is a necessary requirement for population health [22]. He emphasized the moral significance of health, as it contributes significantly to opening up a variety of opportunities for people. Applying Rawls’ theory to the social determinants of health, Daniels advocated that, from a policy perspective, governments should implement policies aimed at equalizing and distributing life opportunities to individuals, e.g. basic education and affordable housing, which could ultimately reduce health inequities [22]. According to the fair equality-of-opportunity principle, therefore, “place” should not determine or restrict an individual’s quality of life. Taking this argument further, we consider in this article how disparities in access to health services in Bangladesh are determined by “place”, and in particular, how inequities in health and access to healthcare are distributed according to the rural–urban divide and, within urban areas, how being located in poor urban settlements limits opportunities for health and quality of life.

## Results

### Ethical dilemmas in the Bangladesh health system: the rural–urban divide

The current health system of Bangladesh is complex, involving several government ministries, the private sector, non-government organizations (NGOs), and development partners, each of which plays a crucial role [23]. The Ministry of Health and Family Welfare (MOHFW) is responsible for formulating health policy and regulation and for ensuring all citizens have access to comprehensive healthcare. The MOHFW supports an extensive country-wide network of health services that includes district hospitals, *upazila* health complexes at the subdistrict level, union health and family welfare centres, and community clinics in rural areas. Although rural health provision is mainly the responsibility of the MOHFW, the urban health system is governed simultaneously by two ministries: the MOHFW and the Ministry of Local Government, Rural Development and Cooperatives (MOLGRDC) [24]. While public tertiary care is provided by the MOHFW in urban areas, according to the local government act primary healthcare (PHC) is the administrative responsibility of local governments through city corporations and municipalities [24]. However, the lack of coordination between the two implementing ministries around service provision, coverage, and referral poses critical challenges to ensuring quality and accessible healthcare, especially for the urban poor.

Due to insufficient human and financial resources at the level of urban local bodies, the MOLGRDC has been providing PHC services in urban areas through an Asian Development Bank (ADB) supported project that contracts out the services to NGOs [25, 26]. As in rural areas, the essential package of services offered is largely focused on maternal, neonatal, and child health, with limited capacity for the prevention and control of emerging and re-emerging diseases or for providing specialized care for men, adolescents, and the elderly [27]. Also, few urban PHC services are offered at convenient times for the working population, and there are no referral linkages from PHC centres to public secondary and tertiary healthcare facilities, as those are governed by the MOHFW [27].

Urban disadvantage and vulnerability lead to various forms of health inequalities. The absence of basic amenities in low-income settlements in urban areas, together with unsanitary environments and overcrowding, creates a vicious cycle of infections, malnutrition, and poor health [10, 28]. The report *State of the World’s Mothers 2015* asserts that the urban slum is one of the worst places to be a mother [29]. Not surprisingly, health indicators are not only far worse in urban slums than in non-slum urban areas, but are well below the national average [10]. While under-five mortality rate is 46 (per 1000 live births) at the national level, the rate is 57 among residents in urban slums [10, 28]. Similarly, neonatal and infant mortality rates in urban slum settlements are double those in non-poor urban areas [30]. Although there are official provisions for antenatal care, skilled birth attendance, and full childhood vaccine coverage from the PHC services, service coverage remains low in urban slums [10, 30]. In the past decade, many infectious diseases have re-emerged in urban areas and are more widespread in urban than in rural areas [31]. Overweight and obesity—risk factors for non-communicable diseases (NCDs)—are also increasing over time even among urban poor women [32]. Hypertension, diabetes mellitus, cancer, and other NCDs are also more common in urban poor communities [33].

Although the informal sector contributes the most to the economy of Bangladesh, workers in this sector, who mostly reside in urban poor communities, experience various forms of exploitation in the workplace but are unable to exercise their right to safe and healthy working conditions. Low pay, long hours, and poor nutrition make many workers vulnerable to health problems, including malnutrition and micronutrient deficiencies [34]. At the same time, unsafe work conditions occasionally lead to disabilities and death from injuries.

Because of inadequate public service provision in urban areas, many urban dwellers seek care from the formal and informal private sectors, which are rapidly increasing in size and importance [35]. More than 70% of the urban poor seek care from the informal private sector (i.e., pharmacies, drug sellers, and traditional healers) as a first point of care [27]. Unregulated over-the-counter drug selling by untrained informal providers is of obvious concern, as is the high cost of formal private sector care, differentially impacting the poorer segment of the population and leading to high out-of-pocket expenditures [36]. Unnecessary diagnostic tests and caesarean sections are also common and impose a substantial economic burden on the poor. High out-of-pocket expenditures for these items may be catastrophic for slum dwellers and poor households, leading to deeper impoverishment [37]. Studies in similar settings showed that the poorest households are the most heavily burdened and often resort to borrowing and selling assets to meet expenses, in the absence of provisions for financial and social protection that would mitigate these impacts [38].

In Bangladesh, physicians’ dual practice in both public hospitals and private clinics leads to frequent absenteeism from public sector services. Unethical interactions between pharmaceutical industries and physicians also lead to negative outcomes that compromise patients’ well-being [39]. Aggressive pharmaceutical promotion poses an ethical threat to physicians’ professionalism by influencing prescribing behaviours that are not in patients’ best interests despite endorsement of 1994 Code of Pharmaceutical Marketing Practices [40]. In the absence of regulation and accountability, serious incidents of medical malpractice and exploitation have been reported, especially in the private sector, that disproportionately affect the poor and less educated. These instances of exploitation, dehumanization, and lack of ethical professionalism in health service delivery are also apparent in clinical and public health research dealing with human subjects [41]. Disadvantaged and illiterate patients with limited access to health information are often not informed of potential risks and adverse effects prior to medical procedures.

A review of bioethics in South Asia further notes distinctions between local practices and Western principles, pertaining to activities such as discussing informed consent, applying norms in clinical decision-making that perpetuate physician paternalism, involving families in decision-making, and providing precise information to patients [42]. The issue of obtaining informed consent is of particular importance in social settings where patients belong to disadvantaged populations [43]. Lack of trust or communication between healthcare providers and patients is another source of stress and anxiety for the urban poor. Due to low educational status and social position, the urban poor are often unable to voice their concerns or understand medical advice. Being unaware of their right to respectful and timely quality care, many of the urban poor are incapable of asserting themselves or demanding quality and fairness [44].

### Ethical obligations of state and non-state actors

As mentioned earlier, the Bangladesh government has committed constitutionally to provide health services to all citizens equally [7]. Bangladesh has been acclaimed by the international community for its significant success in terms of the Millennium Development Goals (MDGs), especially in reducing maternal and child mortality. Recently, Bangladesh has signed the United Nations Sustainable Development Goals (SDGs), which, among many other goals, obliges the country to provide healthcare to all, irrespective of social class, and to make cities and human settlements inclusive, safe, and resilient [45]. Bangladesh has also set the target to achieve universal health coverage (UHC) by 2032 [46]. However, unless action on the social determinants of health is prioritized and good quality service to the underserved segment of the population is ensured, achieving UHC and SDGs will be challenging.

Urban health service delivery is hindered by a lack of clear and functional mechanisms for coordination and planning within and between health and local government ministries, as well as insufficient implementation capacity at the local level [24]. There are both substantial overlap and fragmentation in service delivery, which need to be addressed through better coordination and referral linkages between public and private sectors [24]. Given its constitutional obligation, the government is responsible for the rights to health of its citizens and, by extension, the regulation of both state and non-state actors in health [7]. Towards this goal, the MOHFW has made significant progress in improving accountability and quality of service delivery through the implementation and scale-up of health management information systems (HMIS). However, HMIS data on urban areas are scant, with the exception of large hospitals and donor-funded NGO networks. Data from the private sector are still not transmitted as a regular practice despite that sector’s overwhelming presence in the healthcare system. This is particularly the case among smaller clinics serving poor urban settlements, where capacity and coordination to collect and use routine health information are limited. The recently migrated poor who settle in urban slums are often absent in the system, a situation that jeopardizes their health entitlement [47]. Despite the overwhelming presence of private healthcare service delivery in urban areas, this dimension remains completely undocumented.

On a positive note, the NGO sector has played, and can continue to play, an important role in service delivery in collaboration with government and other stakeholders. In terms of supporting the goal of UHC [48], evidence suggests that urban NGOs contribute importantly to improved coverage, equity, quality of care, and efficiency, although they remain a relatively minor player in the urban healthcare landscape [49]. Also noteworthy, on the other hand, are criticisms of the NGO sector’s susceptibility to donor directives and biomedical approaches that ignore the broader context of development [50]. Evidence of duplication in the sector similarly shows a failure to abide by the Paris Declaration, which emphasizes the coordination of aid in the health sector as an ethical obligation [51].

## Discussion

This paper identifies the emerging health challenges facing the urban poor in Bangladesh as a consequence of rapid and unplanned urbanization and of the absence of coordinated urban health governance in a pluralistic health system. Referring to the Rawlsian theory [21] of “equality of opportunity” to ensure social justice, we have identified the existing ethical dilemmas and moral obligations of the state and non-state sectors in serving the urban poor in Bangladesh.

In his *Theory of Justice*, Rawls put forward the idea that, to ensure social justice, the basic institutions of society need to function in compliance with the principles of justice. Rawls thus placed particular emphasis on the mechanism through which the principles of justice are formulated. His views on “original position” and “veil of ignorance” suggest ways of formulating the principle of justice to support the building of a just society. Rawls’ position was in opposition to classical utilitarianism, which advocates maximum well-being for the maximum number of people. In his view, classical utilitarianism seems to favour the majority over the minority and is ethically unfair, as it results in depriving society’s poorest and weakest of access to basic rights and liberties. Therefore, Rawls argued mainly for a fair distribution of goods. His idea of fairness would see individuals in a society having access to the services they need; in his justice criterion of the “Maximin principle”, Rawls focused particularly on ensuring the well-being of disadvantaged groups in society [21].

In Bangladesh, the MOHFW has achieved notable success in recent years through its sector-wide approach, especially in terms of service delivery in rural areas [52]. However, its stewardship is constrained by a weak legal framework and institutional inadequacies, particularly around regulation and coordination. The government’s capacities to regulate, plan, and provide health services have been further challenged by rapid urbanization [27]. Given that Bangladesh has committed to provide health services for all its citizens and to ensure their right-to-health, decision-makers need to be cognizant of the many ethical dilemmas inherent in the urban health space. In this context, the Rawlsian approach could provide useful guidance for identifying the current status of justice in healthcare policies, especially in terms of how the existing system treats the disadvantaged groups in society. Rawls’ idea of principles of justice stipulates that individuals should not be discriminated against on the basis of their place in society. The urban poor in Bangladesh, however, are facing more daunting challenges in terms of healthcare outcomes in comparison with their rural counterparts. Considering the urban poor as disadvantaged group, the Rawlsian approach, with a view to establishing the fair distribution of goods, would suggest that national health policies and strategies need to incorporate urban health as a distinctive area of focus. In particular, special attention should be given to addressing deep inequities in access to healthcare and reducing the disproportionate exposure of the urban poor to adverse social, economic, and environmental conditions that provoke ill health. Important in this response is recognition of the heterogeneity of the urban poor and the context-specific challenges they face. To improve slum conditions, integrated urban development efforts are especially needed that address issues of housing, tenure security, water and sanitation, green space, education, and healthcare [53].

In Bangladesh, the urban population’s contribution to the Gross Domestic Product (GDP) is estimated as 50% [14]. Urban slum dwellers having migrated from rural areas in search of better economic opportunities are an important part of this economic growth. As a matter of human rights, therefore, slum dwellers should have the same opportunity as others in the country to access basic amenities as full citizens. To achieve this, the State will need to give special attention to policy planning for the poor and disadvantaged in urban Bangladesh. Moreover, there is evidence that, in developing countries, focusing on urbanization in a positive and proactive way can result in a large portion of the urban poor population becoming strong contributors to overall economic growth, rather than being a social burden [53, 54]. In light of these examples, Bangladesh should adopt an inclusive strategy for urban areas targeting the health issues of the urban poor.

In discussions around implementing Rawls’ approach in healthcare, one key observation has been that Rawls did not mention health and the social determinants of health as primary goods. However, Norman Daniels has further enhanced Rawls’ theory to include health services. Starting from a “normal function” premise, Daniels argued that health has a moral significance, in that it opens up other life opportunities to an individual, and a society will be unjust if it allows health inequalities to limit the individual’s basic life opportunities. From this ethical standpoint, Daniels advocated that, apart from the biomedical concept of health, all the social determinants that affect health should be considered for fair distribution according to Rawls’ principle of justice [55]. Daniels’ proposition on social determinants has been helpful in developing specific implementable actions for achieving health that have mitigated the deliberate exclusion of health in Rawls’ theory.

Displacing the biomedical paradigm of health and its focus on the discovery, treatment, and cure of disease and illness, the broader concept of social determinants of health and their contribution to sustainable development and health is widely accepted [56]. The increasing interactions among health, sustainable development, environment, and gender have underscored the importance of the cumulative effect of interventions across various components of society to ensure social justice [57]. In Bangladesh, policy-makers also need to acknowledge that health is not an output of the health sector alone, but an outcome of many other factors and sectors beyond the confines of health services provision. The government of Bangladesh should prioritize a “Health in All Policies” (HiAP) approach to public policies across sectors that systematically takes into account the broader social determinants of health [58]. As theorized by Rawls, this approach will provide the foundation for greater equality of opportunity [21] for the urban poor and disadvantaged and thereby ensure social justice in health and other entitlements. HiAP is particularly important for urban settings, where health problems are concentrated and their determinants are complex. In short, without successful integration and coordination between the health and non-health sectors, the health of the urban poor will not be improved [59, 60].

Investments in social protection and health insurance may reduce out-of-pocket expenditures and help prevent catastrophic medical expenditures, while greater coordination among urban service providers around hours and locations, community outreach, and referral may improve services coverage and use [61]. A strengthened referral system joining different levels of care and spanning private and public sectors is particularly crucial.

Urban health financing is often fragmented because of aid modalities that focus on time-limited projects [58], while fragmentation and poor coordination among implementing agencies in both state and non-state sectors undermine quality, equity and efficiency of health services. These inefficiencies in urban health services provision might be overcome by strengthening governance at both national and local levels. This would require energizing the public health mandates of local governments and ensuring coordination among implementing bodies. While coordination is crucial to address bottlenecks in governance that hinder the availability, accessibility, and utilization of urban health services, it requires policy support at the highest level to be effective [24].

## Conclusion

In sum, health is neither an isolated process output nor the sole concern and responsibility of the health sector. Multiple sectors and actors—including those within national and local governments, private agencies, NGOs, and international partners—need to work together on the multiple social determinants of health. This calls for gaining a deep understanding of the needs of the urban poor through intersectoral forums and then implementing needs-based social interventions.

Although the purpose of this paper is to examine the health of the urban poor from a rights perspective, our major focus has been on the obligations of the supply side, including both the health and related non-health sectors. Government should not only implement regulatory mechanisms to establish the governance of both state and non-state sectors, but also ensure every citizen’s right to obtain good quality healthcare services from all sectors when needed. At the same time, both the state and non-state sectors should ensure a supportive environment is created to address demand-side issues that concern the public, such as improving health literacy and increasing citizens’ awareness of their legal rights and responsibilities. It might also be useful to create a social solidarity mechanism by developing a mutual cooperation system among the urban poor, which could be particularly beneficial during emergency situations often faced by this group. While better coordination is crucial to strengthen the quality, coverage, and equity of urban health services delivery, optimal strategies will be those that take into account the heterogeneous needs of the urban poor and disadvantaged. The particular needs of vulnerable groups such as the extreme poor, women, children, and the disabled, warrant in-depth investigation and the implementation of specialized service delivery approaches to ensure health equity. Decision-makers in Bangladesh should explore policies and programs undertaken in similar settings in the global South and tackle this challenge by identifying best practices and learning from both successes and failures.

Ultimately, progress in the health of the urban poor will depend on how well the Bangladesh government can exercise good governance and ensure accountability. Strong stewardship and commitment to strengthening systems in order to provide equitable and ethical health services for the urban poor is a crucial step towards ensuring all citizens enjoy their right-to-health and reach their full potential.

## Abbreviations

ADB: Asian Development Bank
GDP: Gross domestic product
HiAP: Health in all policies
HMIS: Health management information systems
icddr,b: International centre for diarrhoeal disease research, Bangladesh
ICESCR: International covenant on economic, social and cultural rights
MDGs: Millennium development goals
MOHFW: Ministry of Health and Family Welfare
MOLGRDC: Ministry of Local Government, Rural Development and Cooperatives
NCDs: Non-communicable diseases
NGOs: Non-government organizations
PHC: Primary healthcare
SDGs: Sustainable development goals
UDHR: Universal declaration of human rights
UHC: Universal health coverage
WHO: World Health Organization
